# Update on Drugs to Treat Diabetes

**DOI:** 10.1308/003588412X13171221591213

**Published:** 2012-05

**Authors:** F Game

**Affiliations:** Consultant Diabetologist,Derby Hospitals NHS Foundation TrustUK

## Abstract

Diabetes is a common disease worldwide with a multitude of complications and high mortality. Moreover, its prevalence is increasing and many of our patients will have diabetes. We have known for almost 50 years that patients with diabetes undergo surgical procedures at a higher rate than patients who do not have the condition^1^ and that they spend 45% longer in a hospital bed than patients with diabetes admitted to a medical ward.^2^

In this two-part article, Dr Frances Game introduces us to agents used in diabetes in part 1 and discusses the peri-operative care of diabetic patients in part 2.

Dr Game is a Consultant Diabetologist and Honorary Clinical Associate Professor at Derby Hospitals NHS

Foundation Trust. She is Associate Editor for *Diabetic Medicine* and has been involved in developing national guidelines for patients with diabetes.

References1GallowayJAShumanCRDiabetes and surgery. A study of 667 casesAm J Med196334:1771911394626410.1016/0002-9343(63)90052-42MoghissiESKorytkowskiMTDiNardoMM*et al*
American Association of Clinical Endocrinologists and American Diabetes Association consensus statement on inpatient glycemic controlDiabetes Care2009321,1191,13110.2337/dc09-9029PMC268103919429873

## Question

A 60-year-old, slightly overweight man tells you at a pre-operative consultation that he is on a weekly injection for his diabetes. He thinks it is ‘a sort of insulin’. Is it?

At present, diabetes affects 4–5% of people in the UR and its prevalence is expected to rise by more than 50% over the next decade.^1^ Diabetes related co-morbidities increase the need for surgical procedures and about 10% of patients referred for surgery currently have the disease. Most surgeons will therefore manage patients with the condition on an almost daily basis.

Patients with type 1 diabetes are always treated with insulin. However, type 2 diabetes is a progressive disorder and, as a result, patients who may start with diet and lifestyle modifications will frequently progress to oral hypoglycaemic agents and/or insulin therapy to maintain their glucose control. There is growing pressure to improve the management of these patients and there has been a recent rapid rise in the number of new therapeutic agents licensed for this indication.

## Oral hypoglycaemics

*Biguanides:* These drugs aim to move sugar into cells and most healthcare professionals will be familiar with metformin. They have a low risk of hypoglycaemia but can precipitate lactic acidosis in renal impairment.

*Sulphonylureas:* These drugs stimulate the pancreas to release more insulin. Examples include gliclazide, glibenclamide and glipizide. The risk of hypoglycaemia with these drugs increases with the half-life of the agent.

*Meglitinides:* These drugs work much like sulphonylureas and stimulate the pancreas to release more insulin. They have the potential to cause hypoglycaemia but are shorter acting than sulphonylureas and are therefore often used in the elderly or in those with renal impairment. They should not be used with a sulphonylurea. Examples include nateglinide and repaglinide.

*Thiazolidinediones* (‘glitazones’): These drugs improve the work of insulin on muscle and fat. Pioglitazone is currently the only agent in this class still marketed. There is a low risk of hypoglycaemia although the class effect of fluid retention (leading to unwelcome weight gain for many patients) is well recognised. It is therefore contraindicated for patients with pre-existing heart failure and should be discontinued in patients who develop oedema (including macular oedema). There has been a recent concern about an increase in the risk of bladder cancer in patients on this agent and it has been withdrawn in several countries.

*Dipeptidyl peptidase-4 inhibitors* (‘gliptins’, eg sitagliptin, vildagliptin): Glucagon-like peptide-1 (GLP-1) is a hormone secreted from the small intestine during a meal. It stimulates insulin biosynthesis, inhibits glucagon secretion, slows gastric emptying and reduces appetite, making it an ideal therapeutic target for patients with type 2 diabetes. GLP-1 has a very short half-life due to rapid inactivation by the enzyme dipeptidyl peptidase-4 (DPP-4). Inhibition of DPP-4 with the above agents can therefore lead to potentiation of endogenous GLP-1. The risk of hypoglycaemia is low as the effects of the drugs depend on GLP-1 secretion, which is meal dependent.

## Injectable agents Insulin

AH patients with type 1 diabetes and many with type 2 will be treated with subcutaneously injected insulin. Errors in insulin prescribing are unfortunately very common and insulin has been identified as one of the top five high risk medications in the inpatient environment.^2^ A third of all inpatient medical errors leading to death within 48 hours of the error involve insulin administration.^3^ Confusion often arises from the sheer number of preparations, the administration devices currently available and whether the patient has type 1 diabetes (when insulin should *never*be withheld).

It is the speed of the absorption of insulin from the subcutaneous injection site that differs between the insulin preparations (Table 1). This can be altered by manipulating the amino acid structure of the insulin protein (the insulin analogues) or by crystallising with protamine. Once in the circulation, the half-life of insulin is 5–7 minutes, regardless of the preparation used.

**Table 1:** Insulins

**Type (action)**	**Source**	Name^*^
Short acting (soluble)	Human	Actrapid®
(average onset 30 mlns, peak 2–4 hrs)		Humulin® S Insuman® Rapid
	Pork	Hypurln® porcine neutral
	Beef	Hypurin® bovine neutral
Rapid acting analogues	Analogue	NovoRapid®
(average onset 15 mins, peak 1–2 hrs)		Humalog®
		Apidra®
Intermediate acting (average onset 1–3 hrs, peak 9–12 hrs)	Human	Insulatard®
		Humulin® 1
		Insuman® Basal
	Pork	Hypurin® porcine Isophane
	Beef	Hypurin® bovine Isophane
Long acting analogues (onset 1 hr, even action 24 hrs)	Analogue	Glargine (Lantus®)
		Detemlr (Levemir®)
*Mixtures*
Soluble/lsophane mixes (blphasic)	Human	Humulin® M3 (30/70% mix Humulin® S and Humulin® 1)
		Insuman® Comb 15 (15/85% mix Insuman® soluble and Insuman® Basal)
		Insuman® Comb 25 (25/75% mix Insuman® soluble and Insuman® Basal)
		Insuman® Comb 50 (50/50% mix Insuman® soluble and Insuman® Basal)
	Pork	Hypurin® porcine Isophane 30/70 mix (30/70% mix Hypurin® porcine soluble and porcine Isophane)
Analogue mixes (blphasic)	Analogue/human	NovoMix® 30 (30/70% mix NovoRapid® and Insulatard®)
		Humalog® mix 25 (25/75% mix Humalog® and Humulin® 1)
		Humalog® mix 50 (50/50% mix Humalog® and Humulin® 1)

* Note how the names of different insulins are similar and could lead to drug errors (eg Humalog® and Humalog® mix 50).

Many patients still use pork or beef insulin and the absorption of these is variable. Patients should not therefore be switched to human insulin without careful consideration and monitoring.

The speed of onset of the short acting analogues means that patients must inject just prior to or with a meal and they must not wait the 20–50 minutes that is recommended with standard soluble insulins (eg Actrapid® [Novo Nordisk, Crawley, UR], Humulin® S [Eli Lilly, Windlesham, UR]). If the injection of pre-meal insulin is not timed correctly, then either hyperglycaemia or hypoglycaemia can result, which is a common problem for inpatients with diabetes.

Many patients are taught to ‘count carbohydrates’ and inject a variable amount of insulin around meals and snacks. It is important that they are allowed to continue to do this while in hospital.

Ideally, insulins should be prescribed on a standard chart. The symbol ‘U’ should not be used after the figure denoting the number of units and instead ‘units’ should be written in full (or preferably pre-printed) on the chart to avoid errors. (A ‘U’ can look like a zero if written quickly.)

It is recommended that specific training in the use of insulin for all healthcare professionals should be made mandatory.^2^

It should not be forgotten that insulin is excreted from the kidneys and the duration of action can be increased if the patient has impaired renal function. This can increase the risk of hypoglycaemia.

### GLP-1 agonists

GLP-1 is described above. Analogues of GLP-1 are also available in a subcutaneous injectable form:

> exenatide (twice daily)
> liraglutide (once daily)
> exenatide long acting release (weekly)

They are more potent than the DDP-4 inhibitors and can lead to significant loss of weight. The effect on gastric emptying limits their tolerability for many patients who find the nausea and/or vomiting unacceptable. Hypoglycaemia is uncommon as their action is to enhance postprandial GLP-1 action. An increase in the incidence of acute pancreatitis has been noted in patients on these drugs and it should be discontinued in patients who develop abdominal pain.

In answer to the question at the start of the article, the patient is on a once weekly injectable agent for his diabetes. This is not insulin but a GLP-1 agonist. He will need different peri-operative management to those patients who are on insulin.

**SUMMARY**

> Insulin is still the most common cause of drug errors.
> Insulins with very different actions may have similar names.
> Newer injectable agents are now available. These are not insulins and therefore have a lower risk of hypoglycaemia but can have other adverse effects.
